# Clinical meaningfulness and psychometric robustness of the MG Symptoms PRO scales in clinical trials in adults with myasthenia gravis

**DOI:** 10.3389/fneur.2024.1368525

**Published:** 2024-06-24

**Authors:** Antoine Regnault, Ali A. Habib, Kristin Creel, Henry J. Kaminski, Thomas Morel

**Affiliations:** ^1^Modus Outcomes, A Division of THREAD, Lyon, France; ^2^MDA ALS and Neuromuscular Center, Department of Neurology, University of California, Irvine, Orange, CA, United States; ^3^Department of Neurology and Rehabilitation Medicine, George Washington University, Washington, DC, United States; ^4^UCB Pharma, Brussels, Belgium

**Keywords:** myasthenia gravis (MG), patient reported outcomes (PRO), clinically meaningful change, psychometric analysis, muscle weakness, fatigue

## Abstract

**Objectives:**

The objective of this research was to generate psychometric evidence supporting the myasthenia gravis (MG) symptoms patient-reported outcome (PRO) scales as a fit-for-purpose measure of severity of core symptoms of MG and provide information allowing their meaningful interpretation using data from a phase 3 study in MG.

**Methods:**

Data from the MycarinG study, a phase 3 study of rozanolixizumab in patients with generalized MG who experience moderate to severe symptoms (ClinicalTrials.gov Identifier: NCT03971422) were analyzed with both classical test theory (CTT) and Rasch measurement theory (RMT). Meaningful within-individual change and group-level meaningful change were estimated for three MG Symptoms PRO scales using anchor- and distribution-based methods. Anchor-based methods used patient global impression of severity (PGIS) and change (PGIC) in MG symptoms as anchors.

**Results:**

Good measurement properties of the MG Symptoms PRO scales were shown in the sample of 200 participants: good to excellent reliability (test–retest and internal consistency reliability) and validity (associations between items and scores within the MG Symptoms PRO scales and between the MG Symptoms PRO scores and other clinical outcomes—MG ADL, QMG score, MGC score, and MGFA classes—were as expected); and the items showed good coverage of the continuum and fit to the Rasch model. Triangulation of the anchor- and distribution-based method results led to the definition of clinically meaningful within-patient improvement in scores for Muscle Weakness Fatigability (−16.67), Physical Fatigue (−20.00), and Bulbar Muscle Weakness (−20.00), with associated ranges. Benchmarks are also proposed for the interpretation of group-level results.

**Conclusion:**

The strong psychometric performance of the MG Symptoms PRO scales and the information generated to guide its interpretation supports its use in clinical trials for demonstrating the clinical benefits of new treatments targeting core symptoms of MG (muscle weakness fatigability, physical fatigue, bulbar muscle weakness, respiratory muscle weakness, and ocular muscle weakness).

## Introduction

Myasthenia gravis (MG) is a rare, chronic autoimmune disorder affecting the neuromuscular junction, characterized by fatigable muscle weakness. The muscle groups that are commonly affected are ocular and bulbar muscles as well as limb, neck, and respiratory muscles. A typical characteristic of MG is that weakness tends to increase during periods of activity and improve after rest. In the last decade, there has been a significant increase in therapeutic development for MG and an increasing appreciation that metrics for assessing patient outcomes require improvement (1).

Established outcome measures of the severity of MG include the Quantitative MG (QMG) score (2), the MG-composite (MGC) score (3, 4), and the MG-Activities of Daily Living (MG-ADL) scale (5). The MG Symptoms PRO scales are new patient-reported outcome (PRO) instruments that were developed specifically to measure the core symptoms of MG within clinical trials using sound mixed methods psychometrics. In contrast to other established outcome measures in MG, the MG Symptoms PRO uses a modular approach, as they are five scales independently measuring the severity of five core symptoms of MG: muscle weakness (ocular, bulbar, and respiratory), muscle weakness fatigability (all muscle groups), and physical fatigue. It is the only available MG-specific measure that aims to capture physical fatigue. It was created using the current best standards (6, 7), which emphasize the importance of direct patient input during the development of the instrument. Previous analyses of the MG Symptoms PRO scales demonstrated strong psychometric performance in a sample of 43 participants with generalized MG (8) and highlighted the instrument’s complementarity with the other widely used measures of MG severity, especially its applicability to the milder severity of MG (9).

The use of a PRO measure in clinical trials of new treatments requires solid evidence supporting that it is fit-for-purpose in the given context and that data to guide the interpretation of the results from the measure are available (10, 11). The common approach to support interpretation of a PRO measure is to establish the amount of change in the PRO score that constitutes a clinically meaningful change (12). No information on what constitutes a meaningful change in the MG Symptoms PRO scale scores is available so far, a fact that has hindered its use to date.

The objective of the current study was to confirm the good psychometric performance of the MG Symptoms PRO on a larger sample, using data from the phase 3 MycarinG clinical study testing the safety and efficacy of rozanolixizumab in adults with generalized MG. A second objective was to provide supportive evidence for the interpretation of three of the MG Symptoms PRO scales by providing estimates for clinically meaningful improvement for their scores.

## Methods

### Study design

The MycarinG study was a phase 3, multi-center, randomized, double-blind, placebo-controlled, three-arm study evaluating the efficacy and safety of two doses of rozanolixizumab and placebo in participants with moderate to severe generalized MG (13). Full details on the study design and results of the MycarinG study have been published previously (13). Participants from North America, Europe, and Asia were randomized across three groups: rozanolixizumab 10 mg/kg, 7 mg/kg, or placebo. The randomized, double-blind, placebo-controlled period consisted of a screening period (up to 28 days), a treatment period (6 weeks), and a blinded observation period (8 weeks).

The MG-ADL (5), the QMG score (2), the MGC score (3, 4), and the MG Symptoms PRO scales were collected at screening, baseline, during the treatment period (at Days 8, 15, 29, and 43, plus at Day 22 for the MG-ADL, QMG, and MGC scales), and during the observation period (at Days 71 and 99). Two other PRO instruments were collected at baseline and at the end of the treatment period (Day 43): the MG Impairment Index, a composite instrument of disease severity based on the signs and symptoms of MG (14); and the MG Quality of Life 15-item revised scale, a PRO instrument designed to assess health-related quality of life in patients with MG (15). The MGFA clinical classification was also collected at screening, baseline, end of the treatment period (Day 43), and end of the observation period (Day 99). Finally, two single-item global impression scales of MG symptom severity, a Patient Global Impression of Severity (PGIS), with five response options ranging from “none” to “very severe,” and Patient Global Impression of Change (PGIC), with seven response options ranging from “very much improved” to “very much worse,” were completed by participants to rate the severity of their MG at screening and baseline (for the PGIS only), during the treatment period (at Days 15 and 29), and observation period (at Days 71 and 99). All PRO measures were collected in paper and pencil version.

The MycarinG study (ClinicalTrials.gov Identifier: NCT03971422) was performed in accordance with the principles of the Declaration of Helsinki and the International Conference on Harmonization Guidance for Good Clinical Practice. The study protocol, amendments, and patient-informed consent were reviewed by national, regional, or independent ethics committees or institutional review boards. Written informed consent was obtained from all participants.

### MG Symptoms PRO scales

The MG Symptoms PRO scales are self-administered instruments comprising 42 items in total covering five concepts: muscle weakness Fatigability (nine items), physical fatigue (15 items), bulbar muscle weakness (10 items), ocular muscle weakness (five items), and respiratory muscle weakness (three items). They were developed using a two-step mixed-methods psychometric approach, detailed by Cleanthous et al. (8). The items of the MG Symptoms PRO scales are graded either on a four-point scale for items measuring symptom severity (“none” to “severe”) or on a five-point scale for items measuring symptom frequency (“none of the time” to “all of the time”). The recall period for all items is the previous 7 days. A score is obtained for each MG Symptoms PRO scale by calculating the sum of the individual item scores in each scale and linearly transforming them to a range from 0 to 100 (as per the formula provided in the scoring manual). Hence, the score for each scale ranges from 0 to 100, with higher values indicating more severe symptoms.

The score for each scale is calculated only if more than 70% of the items in the scale are completed. The MG Symptoms PRO scales have been shown to be complementary to the commonly used measures of MG severity and, most specifically, to provide accurate information to discriminate between the milder levels of severity that may not be captured well by these other instruments (9). The MG Symptoms PRO scales have features that make it particularly relevant from a clinical perspective (see Box 1).

BOX 1 **Clinical relevance of MG Symptoms PRO scales.**

The MG Symptoms PRO scales are a new addition to the measurement toolbox available to researchers and clinicians in the MG field. As opposed to the other measures widely used in MG (QMG score, MG-ADL, and MGC score), which are composite indices that summarize the overall MG severity with a single number, the MG Symptoms PRO scales form a modular instrument that provides an independent measurement for the severity of each core symptom of MG. This feature will enable better evaluation of patients with focal weakness (i.e., with localized MG, such as mostly ocular, bulbar or limb). In these patients, an overall measure of severity or impact on activity of daily living may not accurately capture change in the localized area, but this would be reflected in the more specific scores from the MG Symptoms PRO. Hence, the MG Symptoms PRO scales can be used to define more accurate, and interpretable, clinical trial endpoints by providing evidence of the efficacy of the treatment on more specific concepts and it could also define inclusion criteria that would not exclude participants with focal weakness from clinical trials in MG. The modularity of the instrument can also be beneficial to clinical practice, as it could provide useful information on specific symptoms of interest to clinicians. The five scales can be used independently thereby offering flexibility in the use of the instrument: for example, if a patient presents with localized MG affecting mostly (or only) bulbar muscles, the clinician may only use the Bulbar Muscle Weakness scale to have a precise picture of this symptom and monitor it over time, asking only a few well-designed questions.

The MG symptoms PRO scales explicitly include an independent measure of physical fatigue, which is a key complaint of patients with MG and is a manifestation of MG of a different nature to the other core symptoms of the disease that are related to muscle weakness. Having a specific measure of general physical fatigue allows a more accurate and comprehensive clinical picture of the symptomatic experience of patients with MG than the already available outcome measures. This will be useful both for the demonstration of the benefits of new therapies in clinical trials and for the management of patients in clinical practice.

The symptoms evaluated by the MG Symptoms PRO scales cover the full breadth of severity of MG from the mildest to the most severe manifestations. Importantly, as opposed to the other available measures in MG, it was shown to be particularly well-suited to assess people with mild to moderate MG, typically (but not only) through its physical fatigue component, which is a symptom that is particularly experienced by patients who have otherwise mild muscle weakness symptoms but whose life is still impacted by the disease.

However, the MG symptoms PRO scales are, in their current form, not fully fit for use in clinical practice, as they were originally developed for use as clinical trial endpoints. Their current version includes a total of 42 questions, which is too long for use in routine practice, even if only a specific module is used. Further development will be needed to select the relevant items that may inform clinical decisions for the management of individual patients.

### Psychometric and statistical analyses

Psychometric analyses of the MG Symptoms PRO scales were conducted using the data from all participants in the randomized, controlled phase of the study who received at least one dose of rozanolixizumab 10 mg/kg, 7 mg/kg, or placebo. Psychometric analyses were conducted for all five MG Symptoms PRO scales. Meaningful change analyses were only conducted for the Physical Fatigue, Muscle Weakness Fatigability, and Bulbar Muscle Weakness scores; the Ocular Muscle Weakness and Respiratory Muscle Weakness scales were not included in these analyses as they were still considered exploratory at this stage since they included new items that had not been tested before (“Difficulty moving eyes from side to side” and “Difficulty moving eyes up and down” on the Ocular Muscle Weakness scale, and “Difficulty breathing while talking” and “Difficulty breathing when lying down” on the Respiratory Muscle Weakness scale). As psychometric analyses address questions independent from the treatment received, all analyses were conducted blinded from any treatment considerations, with the two rozanolixizumab and placebo treatment groups pooled together.

Analyses were performed in both the classical test theory (CTT) and Rasch measurement theory (RMT) frameworks.

Classical test theory analyses evaluated reliability, construct validity, and the ability of the scales to detect change over time. Reliability coefficients were estimated for all scores using two approaches: internal consistency using Cronbach’s alpha coefficient at baseline and test–retest reliability using interclass correlation coefficients (ICCs) between screening and baseline and between Day 15 and Day 29. Reliability coefficients were interpreted using standard guidelines, with values greater than 0.8 considered adequate (16). Supportive evidence for construct validity was generated by observing whether the PRO score conforms with hypothesized relationships among the items composing the scales or with the scores of other outcome measures or clinical parameters. For this purpose, the correlations across all items composing a scale and between each item and a “corrected score” for the scale to which it belongs (score obtained using all the other items in the scale, i.e., excluding the item under scrutiny) were studied as well as the correlations between the MG Symptoms PRO scale scores and other clinical outcome assessment (COA) scores. All correlations were estimated using the Spearman’s rank order correlation coefficient. The interpretation of the strength of correlation depends on the context. However, no consensus exists for the desirable level of correlations in PRO research, so for the interpretation of these results, we used Cohen’s generic convention (>0.5 = large, between 0.3 and 0.5 = moderate, between 0.1 and 0.3 = small, and ≤ 0.1 = insubstantial) (17). The expected correlation between the MG Symptoms PRO scale scores and the other COA scores were determined depending on whether they reflect the perspective of the same respondent (patient self-report vs. clinician report) and the conceptual overlap of the outcomes being measured. The distribution of the MG Symptoms PRO scale scores was also examined across MGFA classes. Ability to detect change over time was evaluated by calculating standardized effect size (ES) statistics, Kazis’ ES (18), and standardized response mean (SRM) between baseline and Day 29 in subgroups created using PGIC and change in PGIS over the same period. ESs were interpreted according to Cohen’s recommendations (17).

Rasch measurement theory methods use a mathematical model (the Rasch model) to evaluate the legitimacy of summing items to generate measurements (19, 20). The following properties were scrutinized with the generalization of the Rasch model for polytomous items (21, 22): targeting of the items to the sample (a good targeting is achieved when the parameters estimated for the items of a scale match the parameter estimated for the respondents); location of the items along the continuum (the parameter estimated for the items should cover the full continuum without any major gaps); ordering of response categories of the items; item fit [no major deviation between the values expected by the Rasch model and the ones observed, as illustrated by fit statistics—standardized residuals between −2.5 and 2.5 and non-significant chi-square test of fit—and graphical examination of item characteristic curve (21)]; person separation index [PSI—expected to be higher than 0.8 for adequate reliability (16)]; and local dependency between the items (no residual correlation above 0.3).

Statistical analyses were also conducted to generate information supportive of the interpretation of the MG Symptoms PRO Muscle Weakness Fatigability, Physical Fatigue, and Bulbar Muscle Weakness scores. Estimates for meaningful within-patient improvement in scores were obtained by triangulating results from a series of analyses including anchor-based and distribution-based methods, as well as graphical tools (23, 24). Anchor-based methods used anchor instruments, the PGIS and PGIC, to provide information on the meaningful change in the PRO score. The PGIS and PGIC were chosen as anchors since they are conceptually associated with the MG Symptoms PRO scales and are more easily interpreted. The changes in MG Symptoms PRO Muscle Weakness Fatigability, Physical Fatigue, and Bulbar Symptoms scores from baseline to Day 29 (the latest visit of the treatment period where both the PRO scales and the anchors were collected) were described across groups created from the anchors. The distributional statistics of the change in score (median, quartiles, and 10 and 90th percentiles) were compared between participants with no change, one-point improvement, and two-point improvement in PGIS of MG symptoms, as well as between participants who reported stable MG symptoms, “minimally improved” symptoms, and “much improved” symptoms according to the PGIC. The objective was to identify a change in score that discriminated between participants who reported no change according to the anchor and those who reported improvement according to the anchor. Graphical representation of empirical cumulative distribution functions (eCDF) of change in PRO scores according to groups created by the anchors was used to support this discussion. Distribution-based methods based on the statistical distribution of the scores—standardized effect sizes (ES) and standard error of measurement (SEM)—supplemented the estimates from the anchor-based methods. The results of these various approaches were considered together in a qualitative triangulation approach to establish a range of values that can be reasonably considered as meaningful within-patient improvement for each MG Symptoms PRO scale score (10, 23). Finally, the mean change in the MG Symptoms PRO scale scores in subgroups of participants who reported no change, minimal, or moderate improvement offered benchmarks for the interpretation of group-level changes in these scores (e.g., to compare mean change in scores obtained in various treatment groups).

Data analysis was performed using SAS software version 9.4 (SAS Institute Inc., Cary, NC, United States) and RUMM2030 (RUMM Laboratory, Perth, Australia) for the RMT analyses.

## Results

### Sample description

Classical test theory and RMT analyses were conducted using data from 200 participants in the MycarinG study that was fully described earlier (13). The majority were white (68.0%), women (60.5%) and lived in Europe (60.0%) or North America (30.0%). Participants had moderate to severe MG: according to the MGFA classification, 39.0% were categorized as Class II, 57.0% as Class III and 4.0% as Class IV. The description of the MG outcome measures (MG ADL, QMG, MGC scores, and PGIS) at baseline confirmed that participants had moderate to severe MG (Table 1).

**Table 1:** Description of the MycarinG study sample at baseline (*N* = 200).

	MycarinG study sample *N* = 200
MG ADL score, mean (SD)	8.3 (3.4)
QMG score, mean (SD)	15.6 (3.6)
MGC score, mean (SD)	16.0 (6.3)
PGIS—*n* (%)	
None	0 (0.0%)
Mild	39 (19.5%)
Moderate	110 (55.0%)
Severe	45 (22.5%)
Very severe	5 (2.5%)
Missing	1 (0.5%)

Given the study design (and the participant dropouts), 1,329 administrations of the MG Symptoms PRO scales were expected throughout the study follow-up. The full instrument was missing for 7 of the administrations (0.5%); 1,307 administrations (98.3%) were complete (no missing items), and 9 (0.7%) included only one missing item. The missing data were observed across a variety of items of the MG Symptoms PRO scales.

### Psychometric results

#### CTT results

Classical test theory results for all MG Symptoms PRO scales are summarized in Table 2. The Muscle Weakness Fatigability, Physical Fatigue, and the Bulbar Muscle Weakness scales showed good to excellent reliability, with reliability coefficients consistently higher than 0.80 and some greater than 0.90. The patterns of item-to-item and item-to-scale correlations, as well as correlations with other outcome measures (Table 2) and distribution across MGFA classification categories overall corresponded to expectations (Supplementary material 1), which provided solid supportive evidence for the construct validity of the MG Symptoms PRO scales. In particular, the correlations of the MG Symptoms PRO scale scores with the MG-ADL, which also measure MG symptoms from the patient perspective, were higher than the correlations with the QMG and MG-C scores, which are supposed to mainly reflect the clinician perspective at the time of the examination. The higher correlation between the MG Symptoms PRO Bulbar Muscle Weakness score with the MG-C than with the QMG was also expected as, in the MG-C, bulbar symptoms are mostly captured by questions to the patients, very similar to those of the MG-ADL.

**Table 2:** Summary of CTT results.

	Reliability		Construct validity							Ability to detect change over time		
					Correlation with other COAs^3^					Standardized effect size^3^		
	Cronbach’s alpha	ICC^2^	Item-to-Item correlation coefficients	Item-to-Scale correlation coefficients	MGC score	QMG score	MG ADL score	MG II—total score	MG QoL15r score	Worsened patients	Stable patients	Improved patients
	*N*=200^1^	*N* = 116	Range	Range						*N* = 13	*N* = 93	*N* = 78
Muscle weakness fatigability	0.89	0.88	0.35–0.94	0.58–0.71	0.56	0.41	0.68	0.42	0.71	0.31	−0.32	−1.04
Physical fatigue	0.97	0.89	0.48–0.94	0.61–0.89	0.38	0.36	0.49	0.38	0.68	0.30	−0.27	−0.86
Bulbar muscle weakness	0.91	0.83	0.37–0.93	0.55–0.75	0.57	0.26	0.57	0.29	0.53	0.08	−0.30	−0.70
Respiratory muscle weakness	0.88	0.81	0.80–0.86	0.71–0.79	0.41	0.32	0.54	0.28	0.59	0.32	−0.16	−0.49
Ocular muscle weakness	0.83	0.81	0.41–0.92	0.48–0.67	0.33	0.26	0.67	0.41	0.48	−0.05	−0.21	−0.55

^1^Ns for all scales = 200 except Bulbar Muscle Weakness, which is *N* = 197. ^2^Intraclass correlation coefficient between screening and baseline, among stable patients according to PGIS. ^3^Effect size for participants according to change in the PGIS at Day 29. ICC, Intraclass correlation coefficient; MGC, MG composite; QMG, Quantitative MG; MG-ADL, MG activities of daily living; MGII, MG impairment index-total score; MG QOL15r, MG quality of life 14-item revised.

Three findings are worth further elaboration. The first one concerns the Muscle Weakness Fatigability scale: over half of the item-to-item correlations for this scale were lower than what is typically expected to warrant the calculation of a score (between 0.35 and 0.50). The Muscle Weakness Fatigability scale, intended to measure the overall concept of muscle fatigability with usual activities or over the course of the day, regardless of the muscle groups; included by design more heterogeneous items than the other scales (8). The correlations for these items were therefore expected to not be as high as for other scales. For example, the correlations between the item “my voice worsened the longer I was speaking for” and “my legs felt weaker the longer I used them in my usual activities” was the lowest (0.35). This result was expected as these items were designed to capture the expression of a common manifestation, muscle weakness fatigability, but for different muscle groups. The second notable finding concerns the Physical Fatigue scale, which showed correlations lower than 0.50 with all other measures included in the MycarinG study, except the MG-QOL15r. The other measures of overall MG severity do not explicitly capture fatigue; low correlations with these were thus expected. Fatigue is known to be important and impactful for patients, so higher correlation of a fatigue measure with health-related quality of life was expected. Finally, the third notable finding was the fairly low correlation between the MG Symptoms PRO Ocular Muscle Weakness score and the MG-C and QMG, in which ocular muscle symptoms are assessed with ocular exams, while the correlation with MG-ADL, in which the ocular muscle symptoms are patient-reported, was higher. This may suggest that the ocular exam used to capture ocular muscle symptoms in MG (measures of ptosis or diplopia) may not reflect meaningful aspects directly perceived by patients, at least in the format used in the instruments.

An important result of our analyses concerns the ability of the scales to detect change over time. All MG Symptoms PRO muscle scales showed good ability to detect improvement over time, with at least medium standardized ES (>0.5) at Day 29 for participants who improved based on the PGIS. Only the Respiratory Muscle Weakness scale had an ES just slightly below 0.5. The Muscle Weakness Fatigability and Physical Fatigue scales showed large ES for participants who improved according to the PGIS, −1.04 and − 0.86, respectively. The standardized ES for participants improving between baseline and Day 29 were all clearly greater in participants who improved than in stable participants. Only 13 participants (7.0%) were categorized as worsened by the PGIS. The results on ability to detect deterioration are therefore inconclusive.

#### Rasch measurement theory

Rasch measurement theory results for all MG Symptoms PRO scales are summarized in Table 3. Each MG Symptoms PRO scale had adequate to very good targeting, meaning that the range of severity of the items covers the range of severity observed in the study population (Supplementary material 2). However, visual inspection of the person-item threshold distribution of the Bulbar Muscle Weakness and Ocular Muscles Weakness scales showed some gaps in conceptual coverage, suggesting that these two scales may be not as consistently precise over the full range of severity. Additionally, a fair proportion of participants were at the floor of the Respiratory Weakness (32%), Bulbar Weakness (40%), and Ocular Weakness (23%) scales indicating that these symptoms were often not experienced at all by many participants in the overall sample pooling all visits of the study. All items of all scales had ordered category threshold parameters, indicating that the response scales were functioning as expected and that participants were able to accurately discriminate between the response options composing the response scales.

**Table 3:** Summary of RMT results.

	Location parameter (SE)	Standardized fit residuals^2^	Chi-square fit statistics^3^	Local dependency^4^	PSI
**Muscle weakness fatigability^1^**					0.85
35. Legs weakened with longer use	−0.66 (0.03)	−1.00	17.14	Item 34	
34. Arms weakened with longer use	−0.64 (0.03)	−1.79	20.17	Item 35	
38. Vision worsened with longer focusing of eyes	−0.15 (0.03)	**2.53**	12.78		
40. Eyelid drooping worsened with longer focusing of eyes	0.06 (0.03)	**9.16**	**114.62**		
36. Breathing became difficult with longer performance of daily activities	0.08 (0.03)	0.01	10.37		
37. Speech worsened with longer speaking	0.18 (0.03)	**−3.85**	**40.60**	Item 39	
39. Voice worsened with longer speaking	0.30 (0.03)	**−3.67**	**36.03**	Item 37	
41. More difficult to chew at the end of the day	0.36 (0.03)	**−3.46**	**29.74**	Item 42	
42. More difficult to swallow at the end of the day	0.48 (0.03)	−2.49	18.00	Item 41	
**Physical fatigue**					0.96
19. Physically tired	−1.22 (0.04)	0.91	17.63	Item 23	
23. Lack of energy	−0.80 (0.04)	**−3.04**	**38.48**	Item 19	
20. Legs weak	−0.52 (0.04)	0.94	6.77	Item 29	
29. Legs heavy	−0.15 (0.04)	0.69	6.35	Item 20	
22. Arms weak	−0.14 (0.04)	1.65	16.36	Item 31	
28. Physically exhausted	−0.09 (0.04)	**−3.83**	12.77	Item 27	
25. No strength in muscles	−0.06 (0.04)	**−5.79**	23.57		
21. Body could not keep up	−0.02 (0.04)	0.20	10.99		
27. Feel drained	0.00 (0.04)	−1.95	26.75	Item 28	
26. Whole body weak	0.03 (0.04)	**−9.42**	**45.71**		
24. Neck weak	0.31 (0.04)	**16.74**	**487.65**		
31. Arms heavy	0.48 (0.04)	−1.21	18.64	Item 22	
30. Physically hard to get up and start moving	0.57 (0.04)	−1.96	**33.51**		
32. Whole body heavy	0.61 (0.04)	**−7.51**	**41.94**	Item 33	
33. Hard to move body	1.01 (0.04)	**−5.38**	**38.99**	Item 32	
**Bulbar muscle weakness^1^**					0.86
8. Difficulty chewing food	−0.51 (0.05)	**2.53**	19.13		
9. Difficulty swallowing food	−0.28 (0.05)	−0.79	17.57		
7. Weak voice	−0.21 (0.05)	0.42	11.21	Item 6	
12. Nasal voice	−0.03 (0.05)	−2.21	20.08		
14. Slurred speech	0.01 (0.05)	**−6.00**	**46.52**	Item 13	
13. Difficulty pronouncing words	0.04 (0.05)	**−5.47**	**53.92**	Item 14	
6. Hoarse voice	0.04 (0.05)	2.04	9.52	Item 7	
11. Difficulty controlling liquids in mouth	0.28 (0.05)	−0.92	9.22		
15. Drooping around mouth	0.29 (0.05)	**4.44**	**32.95**		
10. Difficulty swallowing liquids	0.39 (0.05)	−1.74	20.48		
**Respiratory muscle weakness^1^**					0.72
16. Difficulty breathing when doing usual activities	−1.13 (0.06)	−0.09	**23.68**		
17. Difficulty breathing while talking^5^	0.55 (0.06)	−0.82	**54.01**		
18. Difficulty breathing while lying down^5^	0.57 (0.06)	0.95	**21.57**		
**Ocular muscle weakness^1^**					0.71
1. Eyelid drooping	−0.66 (0.04)	**5.44**	**36.68**		
2. Double vision	−0.53 (0.04)	0.65	**34.29**		
3. Blurry vision	−0.24 (0.04)	**2.82**	6.26		
4. Difficulty moving eyes side to side^5^	0.67 (0.05)	**−5.95**	**81.80**	Item 5	
5. Difficulty moving eyes up down^5^	0.76 (0.05)	**−4.88**	**69.60**	Item 4	

PSI, Person separation index; DIF, Differential item functioning. ^1^Within each scale, items are ordered according to item location estimates to show the hierarchy revealed by the RMT analysis: first items are reflective of lower symptom severity, last items of higher severity. ^2^Values in bold are outside the range − 2.5/2.5, indicative of possible fit issues (negative value: over-discrimination; positive value: under-discrimination). ^3^Values in bold have *p* value<0.01 after Bonferroni correction, indicative of possible fit issues. ^4^Items with a standardized residual correlation greater than 0.3. ^5^Item added to the scale after cognitive debriefing interview with people with MG.

The Muscle Weakness Fatigability scale showed an item hierarchy (order in which the items were ordered along the continuum) starting with fatigability of limb muscles, fatigability of ocular muscles, fatigability of respiratory muscles, and finally fatigability of bulbar muscles. Five items displayed fit residuals outside the recommended range of −2.5 to +2.5 and four displayed significant chi-square *p* values (Table 3). After graphical examination, only the item “Eyelid drooping worsened with longer focusing of eyes” showed clear under-discrimination: the responses to this item did not change as much as would be expected for participants with different overall muscle weakness fatigability. Three pairs of items (“Arms felt weaker the longer I used them” and “Legs felt weaker the longer I used them”; “Speech worsens the longer I was speaking for” and “Voice worsen the longer I was speaking for”; and “More difficult to chew at the end of the day” and “More difficult to swallow at the end of the day”) had residuals that were correlated >0.30, suggesting that these items may capture common unique information (local dependency) independent from the shared information reflecting muscle weakness fatigability. This shared information may marginally falsely increase the reliability of the scale. Overall, however, the scale had a PSI of 0.85, indicative of good reliability.

The Physical Fatigue scale showed an interpretable item hierarchy, with “Physical tiredness” and “Lack of energy” representing the lowest levels of physical fatigue and the “Whole body heavy” or “Hard to move” characterizing the highest levels. Seven items displayed fit residuals outside the recommended range of −2.5 to +2.5, five of which also displayed significant chi-square *p* values (Table 3). After graphical examination, only the item “Weakness in the neck” showed clear under-discrimination: the responses to this item did not change as much as would be expected for participants with different overall physical fatigue. Five pairs of items (“Physically tired” and “lack of energy”; “Legs weak” and “Legs heavy”; “Arms weak” and “Arms heavy”; “Feel drained” and “Physically exhausted”; “Whole body heavy” and “Hard to move your body”) had residuals that were correlated >0.30, suggesting that these items may capture common unique information (local dependency) independent from the shared information reflecting physical fatigue. The scale had a PSI of 0.96, indicative of excellent reliability.

The Bulbar Muscle Weakness scale showed an item hierarchy ranging from “Difficulty chewing and Swallowing solid food” to “Difficulty swallowing liquids.” Four items displayed fit residuals outside the recommended range of −2.5 to +2.5, with three also displaying significant chi-square *p* values (Table 3). After graphical examination, two items (“Difficulty pronouncing words” and “Slurred speech”) may over discriminate bulbar symptom severity: these items are characteristic of a precise level of bulbar muscle weakness severity but less informative than expected for other levels of severity. Two pairs of items (“Hoarse voice” and “Weak voice”; and “Difficulty pronouncing words” and “Slurred speech”) had residuals that were correlated >0.30, suggesting that these items may capture common unique information (local dependency) independent from the shared information reflecting bulbar muscle weakness. The scale had a PSI of 0.86, indicative of good reliability.

The Respiratory Muscle Weakness and Ocular Muscle Weakness scales both had lower PSIs but were still above 0.7 (0.72 and 0.71, respectively). The Respiratory Muscle Weakness scale had no items with problematic fit or local dependency; the ordering of the three items was meaningful, ranging from “Difficulty breathing when doing usual activities” to “Difficulty breathing when lying down.” The Ocular Muscle Weakness scale had three items with possible misfit: “Eyelid drooping” (under-discrimination), “Difficulty moving eyes from side to side,” and “Difficulty moving eyes up and down” (over-discrimination).

### Determination of clinically meaningful improvement

#### Anchor selection

The correlation between the change in the MG Symptoms PRO scale score between baseline and Day 29 (the latest treatment visit where both the PRO scales and the anchors were collected) and the identified possible anchors, change in PGIS over the same period and PGIC at Day 29, were moderate for Muscle Weakness Fatigability (0.51 and 0.53, respectively) and Physical Fatigue (0.43 and 0.58, respectively) and weaker for Bulbar Muscle Weakness (0.32 and 0.42, respectively). Overall, these correlations justified the use of the PGIS and the PGIC as anchor variables in the anchor-based approach, but the estimates for the Bulbar Muscle Weakness score from this anchor-based analysis should be considered cautiously.

#### Anchor-based analyses

To identify a threshold for meaningful within-patient change for the three MG Symptoms PRO scales, the distribution of change in these scores between baseline and Day 29 was examined across the categories of the main anchor (change in PGIS over the same period) to discern the level of change in score that best discriminate participants with different amount of change in their response to the PGIS (Table 4).

**Table 4:** Summary of anchor- and distribution-based methods.

	Anchor-based method			Distribution-based method	
	Improvement of two points on the PGIS	Improvement of one point on the PGIS	No change on the PGIS		
Muscle weakness fatigability					
*N*	19 (0)	59 (0)	93 (0)	*N*	189
Mean (SD)	−40.20 (23.30)	−19.30 (20.38)	−7.41 (14.64)	SD_BL_	23.73
Median	−38.89	−13.89	−5.56	0.5xSD_BL_	11.87
Q1, Q3	−58.33, −13.89	−25.00, −5.56	−13.89, 0.00	SEM	7.11
P10, P90	−66.67, −11.11	−47.22, 0.00	−27.78, 8.33		
Physical fatigue					
*N*	19 (0)	59 (0)	93 (0)	*N*	189
Mean (SD)	−33.42 (26.08)	−17.26 (18.24)	−7.32 (14.49)	SD_BL_	25.36
Median	−35.00	−13.33	−6.67	0.5xSD_BL_	12.68
Q1, Q3	−56.67, −10.00	−25.00, −5.00	−16.67, 0.00	SEM	4.35
P10, P90	−68.33, 0.00	−41.67, 1.67	−26.67, 7.26		
Bulbar muscle weakness					
*N*	19 (0)	59 (0)	93 (0)	*N*	189
Mean (SD)	−23.41 (24.63)	−11.86 (16.17)	−5.57 (10.99)	SD_BL_	20.78
Median	−23.33	−10.00	−3.33	0.5xSD_BL_	10.39
Q1, Q3	−36.67, −6.67	−20.00, 0.00	−10.00, 0.00	SEM	6.88
P10, P90	−58.15, 0.00	−36.67, 3.33	−20.00, 3.33		

SD, Standard deviation; Q1, first quartile (25th percentile); Q3, third quartile (75th percentile); P10, 10th percentile; P90, 90th percentile; SD_BL_, Standard deviation at baseline; SEM, Standard error of measurement.

For Muscle Weakness Fatigability, 75% of participants with no change in PGIS had a decrease in score of less than −13.89 or an increase in score (Q1), while half (median) of those who had a one-point improvement on the PGIS and 75% (Q3) of those who had a two-point improvement had a decrease in score of more than this value. For Physical Fatigue, 75% (Q1) of participants with no change in PGIS had a decrease in score of less than −16.67 points or an increase in score, while half (median) who had a one-point improvement on the PGIS (median) had a decrease in score of more than −13.33 and 75% (Q1) of those who had a two-point improvement had a decrease in score of more than −10.00. For Bulbar Muscle Weakness, 75% (Q1) of participants with no change in PGIS had a decrease in score of less than −10.00 points or an increase in score, while half (median) of those who had a one-point improvement on the PGIS had a decrease in score of more than −10.00 and 75% (Q1) of those who had a two-point improvement had a decrease in score of more than −6.67.

#### Clinically meaningful improvement in muscle weakness fatigability

**Figure 1:**
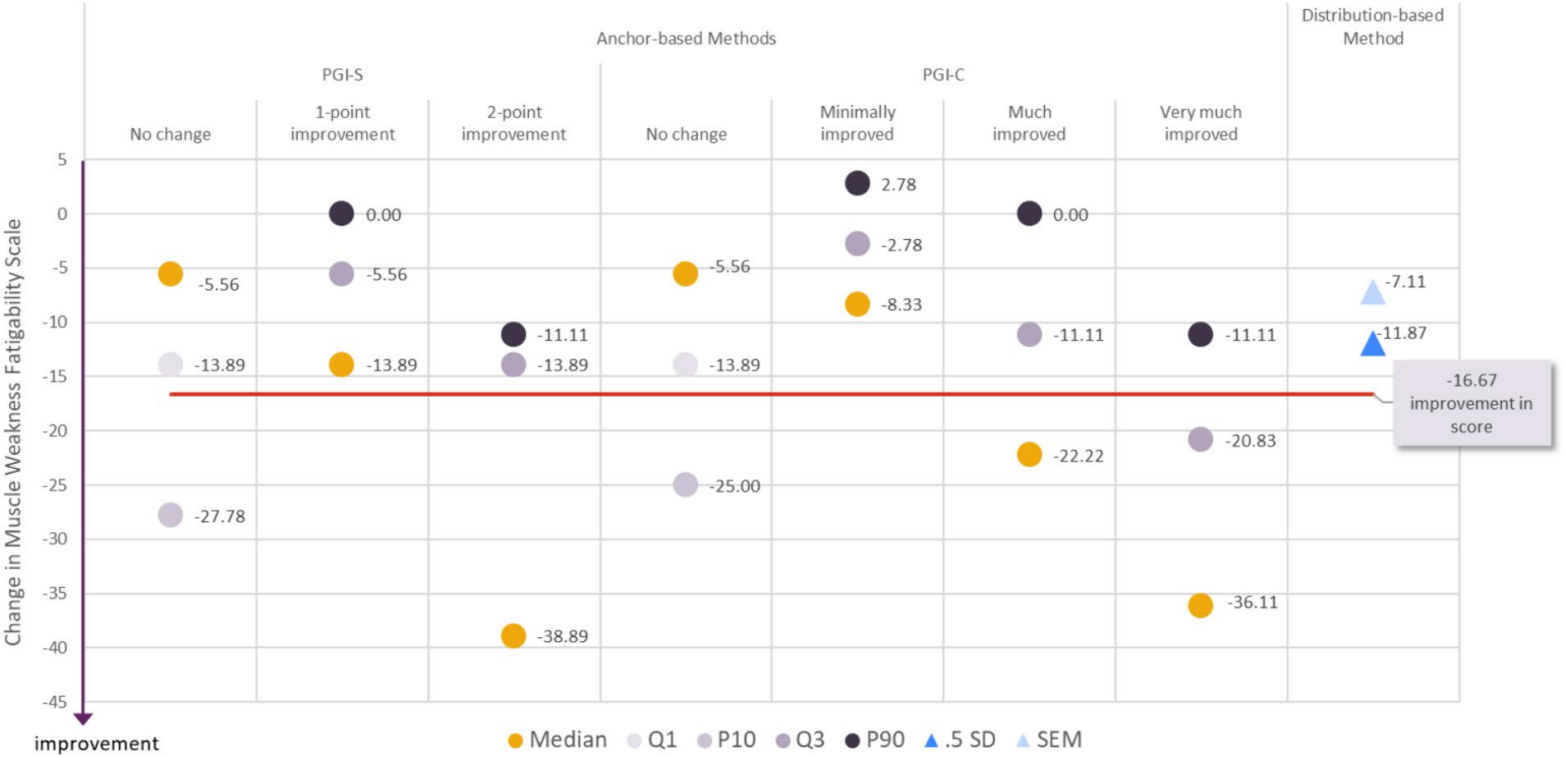
Visualization of estimates from anchor-based and distribution-based analyses used to determine meaningful within-patient change for improvement for the MG Symptoms PRO Muscle Weakness Fatigability score in the MycarinG study.

Variability around what constitutes a meaningful change in a PRO score is a given, since it is dependent on context of use, patient population, and study sample. For these reasons, our proposed estimates for meaningful within-patient improvement in the MG Symptoms PRO scores come with a range of reasonable values. These estimates capitalize on all results from both anchor-based methods and distribution-based methods described above (and referred to as “triangulation”). For the MG Symptoms PRO Muscle Weakness Fatigability score, a decrease of −16.67 points or more appeared to constitute a fair estimate for meaningful within-patient improvement based on the full results observed in our sample (Figure 1). To achieve a change in score of −16.67 points, six items out of nine should report improvement of at least one category (or fewer items reporting improvement of two categories or more). Only 20.4% (19/93) of participants who did not report any change in the PGIS between baseline and Day 29 had a decrease in their MG Symptom PRO Muscle Weakness Fatigability score of more than −16.67 points, while 45.8% (27/59) of participants who reported an improvement of one category in the PGIS and 73.7% (14/19) of participants with an improvement of two categories in PGIS had a decrease in the MG Symptoms PRO Muscle Weakness Fatigability score greater than −16.67.

A range of reasonable values for meaningful within-patient improvement in the MG Symptoms PRO Muscle Weakness Fatigability score includes a decrease as small as −13.89 (corresponding to a decrease of five points in the raw score) and as high as −27.78 points (corresponding to a decrease of 10 points in the raw score).

The difference in mean change in the Muscle Weakness Fatigability score between participants with a 1-point improvement on the PGIS and those with no change on the PGIS was −11.90 points, and between participants with a one- or two-point improvement on the PGIS and those with no change on the PGIS was −16.99 points. Both differences are greater than the results of distribution-based estimates of half a standard deviation (11.87) and SEM (7.11). Those values can therefore be used as benchmark to interpret differences in mean Muscle Weakness Fatigability score observed in group of patients (e.g., between treatment groups of clinical trials).

#### Clinically meaningful improvement in physical fatigue

**Figure 2:**
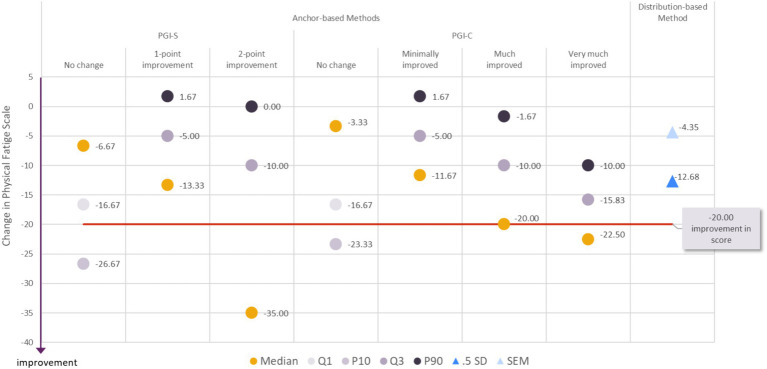
Visualization of estimates from anchor-based and distribution-based analyses used to determine meaningful within-patient change for improvement for the MG Symptoms PRO Physical Fatigue score in the MycarinG study.

As described for the Muscle Weakness Fatigability score above, we propose an estimate for the clinically meaningful within-patient improvement in the Physical Fatigue score associated with a range of reasonable values. When considering all the results (from anchor-based methods and distribution-based methods) together (“triangulation”), it appeared that a decrease of −20.00 points or more in the MG Symptoms PRO Physical Fatigue score would constitute a fair estimate for meaningful within-patient improvement (Figure 2). To achieve a change in score of −20.00 points, 12 items out of 15 should report improvement of at least one category (or fewer items reporting improvement of two categories or more). Only 20.4% (19/93) of participants who did not report any change in the PGIS between baseline and Day 29 had a decrease in their MG Symptom PRO Physical Fatigue score of more than −20.00 points, while 42.4% (25/59) of participants who reported an improvement of one category in the PGIS and 57.9% (11/19) of participants with an improvement of two categories in PGIS had a decrease in the MG Symptoms PRO Physical Fatigue score greater than −20.00.

A range of reasonable values for meaningful within-patient improvement includes a decrease as small as −16.67 (corresponding to a decrease of 10 points in the raw score) to a decrease as high as −26.67 points in the MG Symptoms Physical Fatigue score (corresponding to a decrease of 16 points in the raw score).

The difference in mean change in the Physical Fatigue score was −9.94 points between participants with a one-point improvement on the PGIS and those with no change on the PGIS, and − 13.87 points between participants with a one- or two-point improvement on the PGIS and those with no change on the PGIS. Both differences were greater than SEM (4.35), but smaller than half a standard deviation (12.68). Given the difference between the two distribution-based estimates, and the absence of strong consensus on the most robust distribution-based method, those values may nonetheless be used as a benchmark to interpret differences in mean Muscle Physical Fatigue score observed in group of patients (e.g., between treatment groups of clinical trials).

#### Clinically meaningful improvement in bulbar muscle weakness

**Figure 3:**
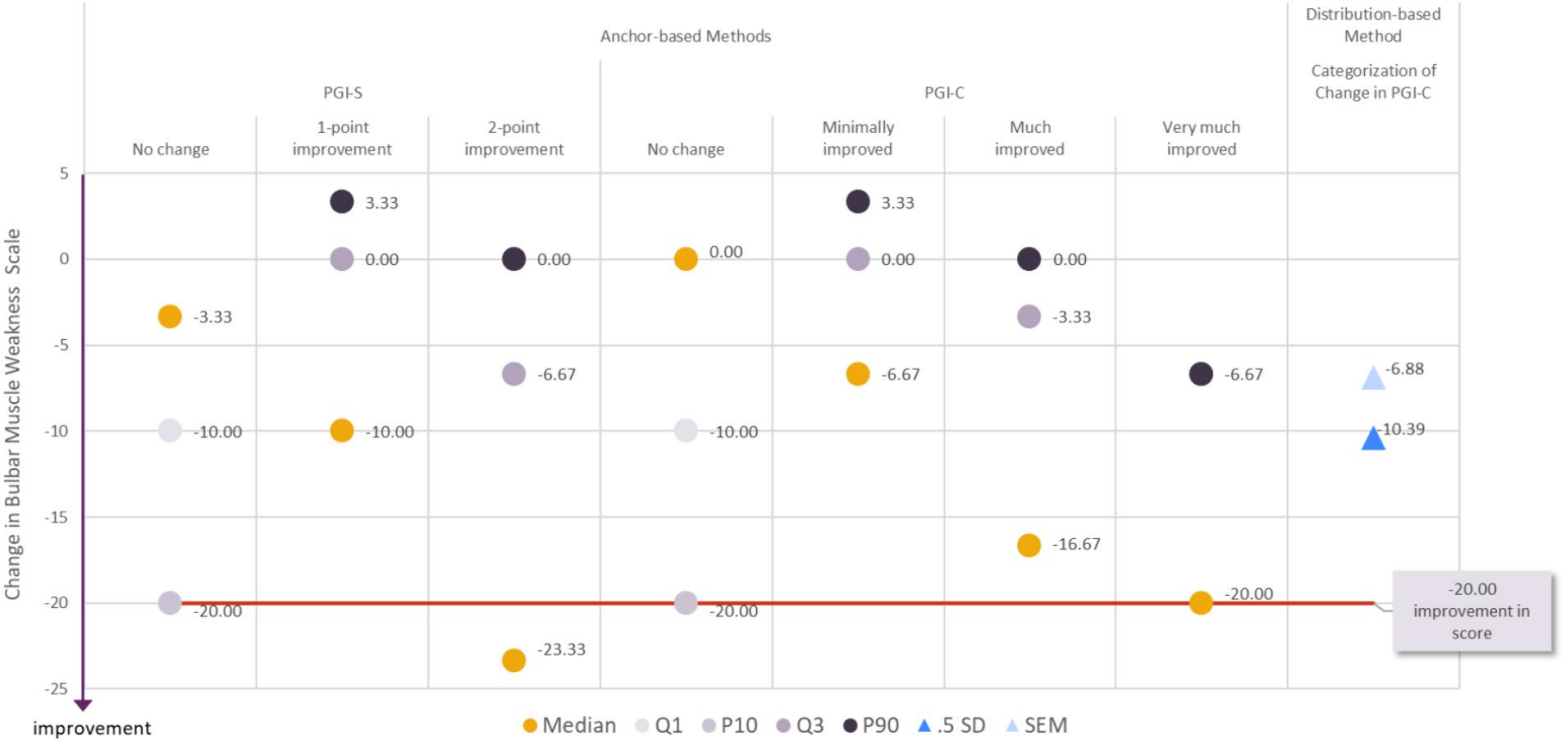
Visualization of estimates from anchor-based and distribution-based analyses used to determine meaningful within-patient change for improvement for the MG Symptoms PRO Bulbar Muscle Weakness score in the MycarinG study.

As described for the Muscle Weakness Fatigability score above, we propose an estimate for the clinically meaningful within-patient improvement in the Bulbar Muscle Weakness score associated with a range of reasonable values. When considering all the results together, it appeared that a decrease of −20.00 points or more in the MG Symptoms PRO Bulbar Muscle Weakness score would constitute a fair estimate for meaningful within-patient improvement (Figure 3). To achieve a change in score of −20 points, six items out of 10 should report improvement of at least one category (or fewer items reporting improvement of two categories or more). Only 14% (13/93) of the participants who did not report any change in the PGIS between baseline and Day 29 had a decrease in their MG Symptom PRO Bulbar Muscle Weakness score of more than −20.00 points while 28.8% (17/59) of the participants who reported an improvement of one category in the PGIS and 63.2 percent (12/19) of the participants with an improvement of two categories in PGIS had a decrease in the MG Symptoms PRO Bulbar Muscle Weakness score greater than −20.00 points.

A range of reasonable values for meaningful within-patient improvement includes a decrease as small as −16.67 (corresponding to a decrease of five points in the raw score) to as high as −26.67 points in the MG Symptoms PRO Bulbar Muscle Weakness score (corresponding to a decrease of eight points in the raw score).

The difference in mean change in Bulbar Muscle Weakness score was −6.29 points between participants with a one-point improvement on the PGIS and those with no change on the PGIS, and − 9.10 points between participants with a one- or two-point improvement on the PGIS and those with no change on the PGIS. These differences did not systematically reach the level of change based on distribution-based estimates of half a standard deviation (10.39) and SEM (6.88). Therefore, while they can be tentatively considered as a benchmark, these values should be used with caution when interpreting differences in mean Bulbar Muscle Weakness score observed in group of patients (e.g., between treatment groups of clinical trials).

The information generated by our analyses to guide the interpretation of the MG Symptoms PRO Muscle Weakness Fatigability, Physical Fatigue, and Bulbar Muscle Weakness scores, both in terms of clinically meaningful within-patient improvement and benchmark for group-level comparison, is summarized in Table 5.

**Table 5:** Summary of data to inform interpretation of MG Symptoms PRO scores as derived from the MycarinG study.

	Meaningful within-patient improvement estimates		Benchmark for group-level comparison (difference in mean change in score)	
	Reference value	Range of reasonable values	One-point improvement vs. stable	One or two-point improvement vs. stable
Muscle weakness fatigability	−16.67	−13.89 to −27.78	−11.90	−16.99
Physical fatigue	−20.00	−16.67 to −26.67	−9.94	−13.87
Bulbar muscle weakness	−20.00	−16.67 to −26.67	−6.29^*^	−9.10^*^

^*^Values to be used with caution as lower than distribution-based method estimates.

## Discussion

Our psychometric analyses of the data collected in the MycarinG study provided additional evidence, from a larger sample, consolidating the demonstration that all five MG Symptoms PRO scales are adequate measures of the severity of cardinal symptoms of MG. They also provide useful information for the interpretation of three scales of the instrument (Muscle Weakness Fatigability, Physical Fatigue, and Bulbar Muscle Weakness scales) both at the individual and group levels.

The MG Symptoms PRO scales showed strong results in both the CTT and RMT paradigms, confirming the good psychometric results already published for the instrument (8). The extremely low amount of missing data (98.3% of assessments having no missing items) indicates that the MG Symptoms PRO scales, despite their 42 items, were very well accepted by the study participants, which corroborates previous PRO development efforts to ensure patient-driven content validity (8). The reliability coefficients for MG Symptoms PRO scale scores obtained in this sample were very similar to those previously reported, confirming their good reliability. The reliability of the new ocular muscle weakness and respiratory muscle weakness scores, which included additional items compared to the versions previously reported, were improved, confirming the appropriateness of adding these items to create better measures. The pattern of correlations between the MG Symptoms PRO scales and the MG-ADL, MGC, and QMG was very similar to what was reported previously. The correlations between the Muscle Weakness Fatigability and Bulbar Muscle weakness with these outcome measures were moderate. The Physical Fatigue score had lower correlations, especially with the MGC and QMG scores, which was expected as these instruments do not directly address fatigue. The scores were also distributed as expected across MGFA classes. The RMT results were also very consistent in the two samples, with similar targeting for each scale. The ordering of the items over the severity continuums observed in the two analyses were overall interpretable and consistent, with the notable exception of the Bulbar Muscle Weakness score. For this score, both the items related to voice and those related to swallowing function were not ordered similarly in the two analyses. Additionally, the interpretation of the ordering of these items over the continuum of severity of bulbar muscle weakness was not straightforward: while speech-related symptoms could be expected to reflect lower severity than those related to swallowing, they were not necessarily the ones located toward the less severe end of the continuum. These aspects related to the item distribution over the continuum of bulbar muscle weakness severity suggest that this score may deserve further investigation in the future. Nonetheless, the good psychometric performance previously observed during the development of the MG Symptoms PRO scales was confirmed overall by our analyses, in a much larger sample of patients with MG. The findings should therefore instill greater confidence in the use of the instrument in future clinical trials in MG.

Beyond the confirmation of the early psychometric results, the analyses of the data from the MycarinG study provided additional knowledge on the MG Symptoms PRO scales. The study design—specifically the collection of variables that constituted good anchor variables, such as the PGIS and PGIC—facilitated the generation of additional evidence on the longitudinal psychometric properties of the MG Symptoms PRO scales, evidence that could not be evaluated with previous data. More robust estimates of test–retest reliability could be obtained using the PGIS to define stability between the two assessments. The PGIS and PGIC allowed participants to be categorized depending on whether their MG symptoms improved, were stable, or worsened, thus permitting evaluation of the ability of the MG Symptoms PRO scales to detect change over time. These results demonstrate the viability of the instrument in capturing improvement in MG symptoms, as participants who reported overall improvement in their symptoms also had high improvement in the MG Symptoms PRO scale scores. Finally, and most importantly, our analyses provided reference values to guide the interpretation of the change in three MG Symptoms PRO scale scores both at the individual level (meaningful within-patient improvement) and at the group-level. This information will be critical when considering clinical trial data from these scales.

Ascertaining meaningful change in PRO scores to guide their interpretation is a critical but challenging endeavor. Triangulation of anchor-based and distribution-based methods is typically recommended in this context, with a primary emphasis placed on anchor-based methods (10, 11, 24). But the application of these methods in practice requires careful specification, with many different decisions to be made (which anchors to use, which statistic to consider, etc.), and the detail of this specification still largely varies in the literature (25, 26). PRO scores can be used in various contexts, which can explain the different methodological approaches. They can be used to make inferences concerning individuals (typically in a daily clinical practice context) or to make inferences concerning groups (typically in clinical research) (12). And, even in the context of clinical trials, the interpretation of the PRO score in clinical trials may be done at the group level (comparing mean changes in score between groups) or at the individual patient level (responder analysis comparing the change in score observed for each participant to a threshold defining whether they experienced a meaningful improvement or not). Establishing meaningful change in PRO scores at the group- or individual-level is therefore an important distinction, but the methods for each of these settings are not yet settled (27, 28). In our analyses, we produced both estimates of meaningful within-patient improvement in the MG Symptoms PRO scale scores and benchmarks to support the interpretation of change in score at the group-level (i.e., mean changes in score). The meaningful within-patient improvement estimates were obtained through a triangulation exercise considering a variety of results, but primarily driven by finding values that discriminated between patients who reported improvement in their symptoms and those who did not. The benchmark for group-level interpretation was obtained by comparing mean changes observed in groups of participants. These results constitute a first set of references for the interpretation of the MG Symptoms PRO scale scores, which will need to be confirmed in future research, especially if the scales are used in a different context (e.g., different target populations) than the MycarinG study. It is for this reason that we proposed a range of reasonable values for meaningful within-patient improvement based on our analyses.

A limitation of our research was that all the analyses were conducted in a sample of patients from a phase 3 clinical trial with generalized MG who experience moderate to severe symptoms. This sample is probably more homogeneous than a “real life” MG population. Therefore, while the results clearly support the use of the MG Symptoms PRO scales in this specific context of the trial, questions remain whether this conclusion may be extended to a wider patient population, especially for groups of patients that were not represented in the sample, such as those with ocular MG only. Another limitation was that our results for Ocular Muscle Weakness and Respiratory Muscle Weakness scores were only partial at this stage: no analysis of meaningful change was conducted for these scores. Further research, with more data, will be needed to consolidate the evidence supporting these two scales.

Future research on the MG symptoms PRO scales may involve exploring various outstanding questions. First, the Muscle Weakness Fatigability, Physical Fatigue, Bulbar Muscle Weakness scales include 9, 15 and 10 items, respectively. In contexts requiring rapid completion of the scale (e.g., registry studies, use in routine clinical practice), the scales may be considered too long. The results of our RMT analyses showed some deviations of a few items from the Rasch model that would therefore constitute possible candidate for future item reduction. Importantly, such item reduction should not be based on these quantitative data only but should also factor in qualitative research findings to make sure that the content validity of the MG Symptoms PRO is preserved. The feasibility of constructing short forms for these scales, ideally without damaging their measurement properties, could be explored. This further development of the MG Symptoms PRO scales would be integral to adapting the instrument for wider application in the context of clinical practice. Beyond the need for shorter versions of the scale, this research would require careful design considering the types of critical decisions that could be informed by the MG Symptoms PRO scales, and the expected features for a tool to be used in this specific context. Finally, the MG Symptoms PRO scales were designed from their inception in the RMT framework, which offers additional opportunities to support the interpretation of PRO scales (29), capitalizing on the hierarchy of items provided by the Rasch model. There is therefore the possibility of further exploration of new ways of interpreting MG Symptoms PRO scales using its underlying item hierarchy.

In conclusion, the cumulative body of evidence on the MG Symptoms PRO scales supports their use as fit-for-purpose measures of the core symptoms of MG (muscle weakness fatigability, physical fatigue, bulbar muscle weakness, respiratory muscle weakness, and ocular muscle weakness) in clinical trials. Most importantly, reference values for meaningful change are now available to guide their interpretation at both the individual and group levels.

## Data availability statement

The datasets presented in this article are not readily available however underlying data from this manuscript may be requested by qualified researchers 6 months after rozanolixizumab approval in the United States and/or Europe, or global development is discontinued, and 18 months after trial completion. Investigators may request access to anonymized individual patient-level data and redacted trial documents which may include: analysis-ready datasets, study protocol, annotated case report form, statistical analysis plan, dataset specifications, and clinical study report. Prior to use of the data, proposals need to be approved by an independent review panel at www.Vivli.org and a signed data sharing agreement will need to be executed. All documents are available in English only, for a pre-specified time, typically 12 months, on a password protected portal. Requests to access the datasets should be directed to www.Vivli.org.

## Ethics statement

The studies involving humans were approved by each one of the 81 centers in the study (the full list is available as a Supplemental Materials) with their own ethical approval process. The studies were conducted in accordance with the local legislation and institutional requirements. The participants provided their written informed consent to participate in this study. Written informed consent was obtained from the participants for the publication of study data, including those in this article.

## Author contributions

AR: Writing – review & editing, Writing – original draft. AH: Writing – review & editing, Writing – original draft. KC: Writing – review & editing, Writing – original draft. HK: Writing – review & editing, Writing – original draft. TM: Writing – review & editing, Writing – original draft.
