# Supplementary material for: Clinical meaningfulness and psychometric robustness of the MG Symptoms PRO scales in clinical trials in adults with myasthenia gravis
Source: Front Neurol. 2024 Jun 24;15:1368525. doi: 10.3389/fneur.2024.1368525 (PMC11229520; doi:10.3389/fneur.2024.1368525)
Supplement: Supplementary file 1 [file Data_Sheet_1.docx]

Supplemental Material 1: Description of MG Symptoms PRO scale score per MGFA class

|  | MGFA Classification | | | | | |
| --- | --- | --- | --- | --- | --- | --- |
| Variable | Class IIa N=37 | Class IIb N=41 | Class IIIa N=75 | Class IIIb N=39 | Class IVa N=7 | Class IVb N=1 |
| Muscle Weakness Fatigability Scale |  |  |  |  |  |  |
| n (missing) | 37 (0) | 41 (0) | 75 (0) | 39 (0) | 7 (0) | 1 (0) |
| Mean (SD) | 37.01 (21.56) | 39.70 (22.74) | 48.07 (22.59) | 51.42 (26.64) | 60.71 (15.83) | 75.00 (.) |
| Median | 36.11 | 36.11 | 50.00 | 50.00 | 66.67 | 75.00 |
| Q1, Q3 | 22.22, 50.00 | 19.44, 55.56 | 27.78, 63.89 | 33.33, 69.44 | 55.56, 69.44 | 75.00, 75.00 |
| Min, Max | 2.78, 83.33 | 8.33, 86.11 | 8.33, 100.00 | 8.33, 100.00 | 27.78, 75.00 | 75.00, 75.00 |
|  |  |  |  |  |  |  |
| Physical Fatigue Scale |  |  |  |  |  |  |
| n (missing) | 37 (0) | 41 (0) | 75 (0) | 39 (0) | 7 (0) | 1 (0) |
| Mean (SD) | 44.05 (23.45) | 40.89 (29.82) | 54.13 (20.91) | 49.87 (27.03) | 73.81 (20.20) | 61.67 (.) |
| Median | 41.67 | 30.00 | 55.00 | 50.00 | 75.00 | 61.67 |
| Q1, Q3 | 25.00, 53.33 | 15.00, 66.67 | 40.00, 68.33 | 28.33, 61.67 | 55.00, 93.33 | 61.67, 61.67 |
| Min, Max | 0.00, 96.67 | 1.67, 100.00 | 10.00, 98.33 | 3.33, 100.00 | 50.00, 100.00 | 61.67, 61.67 |
|  |  |  |  |  |  |  |
| Bulbar Muscle Weakness Scale |  |  |  |  |  |  |
| n (missing) | 37 (0) | 41 (0) | 75 (0) | 39 (0) | 7 (0) | 1 (0) |
| Mean (SD) | 19.18 (15.34) | 24.43 (22.41) | 25.64 (19.48) | 35.21 (20.62) | 33.33 (22.93) | 93.33 (.) |
| Median | 20.00 | 20.00 | 26.67 | 33.33 | 33.33 | 93.33 |
| Q1, Q3 | 6.67, 30.00 | 3.33, 33.33 | 10.00, 36.67 | 16.67, 50.00 | 10.00, 56.67 | 93.33, 93.33 |
| Min, Max | 0.00, 63.33 | 0.00, 81.48 | 0.00, 80.00 | 3.33, 83.33 | 6.67, 63.33 | 93.33, 93.33 |
|  |  |  |  |  |  |  |

|  | MGFA Classification | | | | | |
| --- | --- | --- | --- | --- | --- | --- |
| Variable | Class IIa N=37 | Class IIb N=41 | Class IIIa N=75 | Class IIIb N=39 | Class IVa N=7 | Class IVb N=1 |
| Respiratory Muscle Weakness Scale |  |  |  |  |  |  |
| n (missing) | 37 (0) | 41 (0) | 75 (0) | 39 (0) | 7 (0) | 1 (0) |
| Mean (SD) | 19.22 (22.63) | 22.49 (22.97) | 29.78 (24.79) | 39.60 (30.67) | 60.32 (31.98) | 44.44 (.) |
| Median | 11.11 | 22.22 | 22.22 | 33.33 | 66.67 | 44.44 |
| Q1, Q3 | 0.00, 33.33 | 0.00, 33.33 | 11.11, 44.44 | 11.11, 66.67 | 33.33, 88.89 | 44.44, 44.44 |
| Min, Max | 0.00, 66.67 | 0.00, 100.00 | 0.00, 100.00 | 0.00, 100.00 | 11.11, 100.00 | 44.44, 44.44 |
|  |  |  |  |  |  |  |
| Ocular Muscle Weakness Scale |  |  |  |  |  |  |
| n (missing) | 37 (0) | 41 (0) | 75 (0) | 39 (0) | 7 (0) | 1 (0) |
| Mean (SD) | 28.29 (22.79) | 31.71 (22.94) | 32.80 (21.53) | 35.04 (26.08) | 44.76 (26.86) | 73.33 (.) |
| Median | 26.67 | 26.67 | 26.67 | 33.33 | 60.00 | 73.33 |
| Q1, Q3 | 6.67, 40.00 | 20.00, 46.67 | 13.33, 46.67 | 13.33, 53.33 | 13.33, 66.67 | 73.33, 73.33 |
| Min, Max | 0.00, 93.33 | 0.00, 93.33 | 0.00, 93.33 | 0.00, 86.67 | 6.67, 73.33 | 73.33, 73.33 |
|  |  |  |  |  |  |  |
