# Supplementary material for: Clinical meaningfulness and psychometric robustness of the MG Symptoms PRO scales in clinical trials in adults with myasthenia gravis
Source: Front Neurol. 2024 Jun 24;15:1368525. doi: 10.3389/fneur.2024.1368525 (PMC11229520; doi:10.3389/fneur.2024.1368525)
Supplement: Supplementary file 2 [file Data_Sheet_2.docx]

Supplemental Material 2: Targeting of the MG Symptoms PRO scales

**
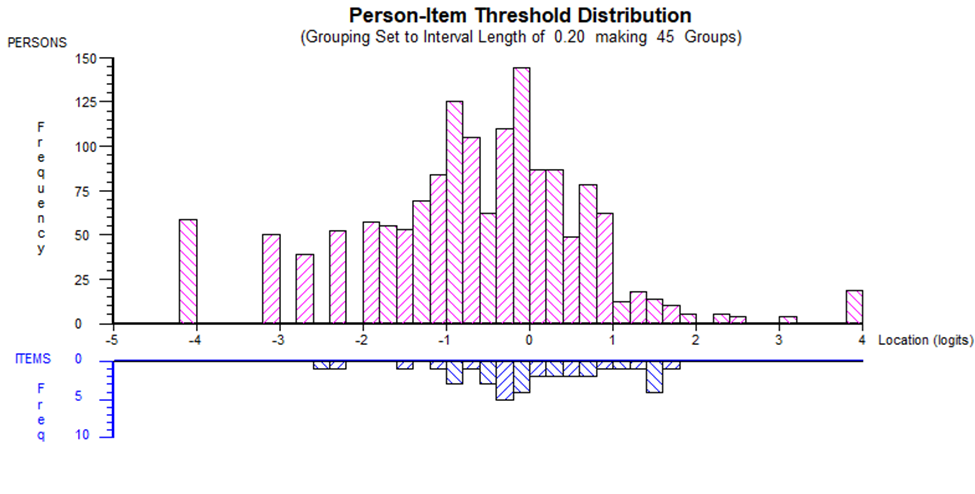
**

Figure 1: MG Symptoms PRO Muscle Weakness Fatigability Targeting (all visits pooled) in the MG0003 study (Safety Sample)

Legend: This figure shows the distribution of person measurements (upper histogram) against the distribution of the item threshold locations (lower histogram) on the Muscle Weakness Fatigability continuum. Here, the lower histogram (blue bars) shows the distribution of item thresholds which represent the boundaries between adjacent response categories.

**
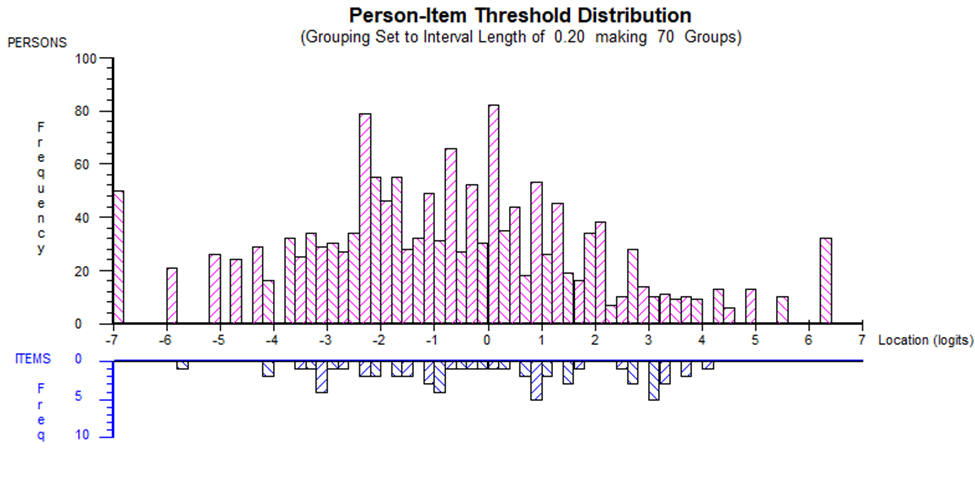
**

Figure 2: MG Symptoms Physical Fatigue Targeting (all visits pooled) in the MG0003 study (Safety Sample)

Legend: This figure shows the distribution of person measurements (upper histogram) against the distribution of the item threshold locations (lower histogram) on the Physical Fatigue continuum. Here, the lower histogram (blue bars) shows the distribution of item thresholds which represent the boundaries between adjacent response categories

**
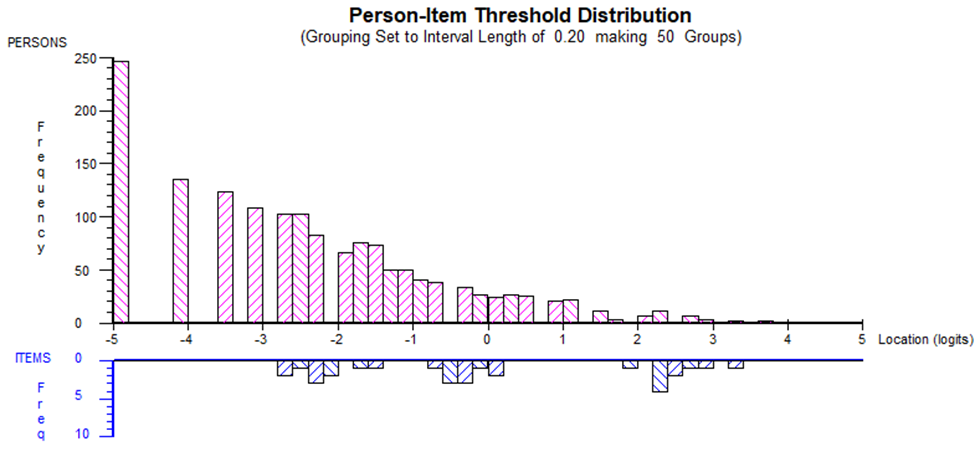
**

Figure 3: MG Symptoms Bulbar Muscle Weakness Targeting (all visits pooled) for the MG0003 study (Safety Sample)

Legend: This figure shows the distribution of person measurements (upper histogram) against the distribution of the item threshold locations (lower histogram) on the Bulbar Muscle Weakness continuum. Here, the lower histogram (blue bars) shows the distribution of item thresholds which represent the boundaries between adjacent response categories.

**
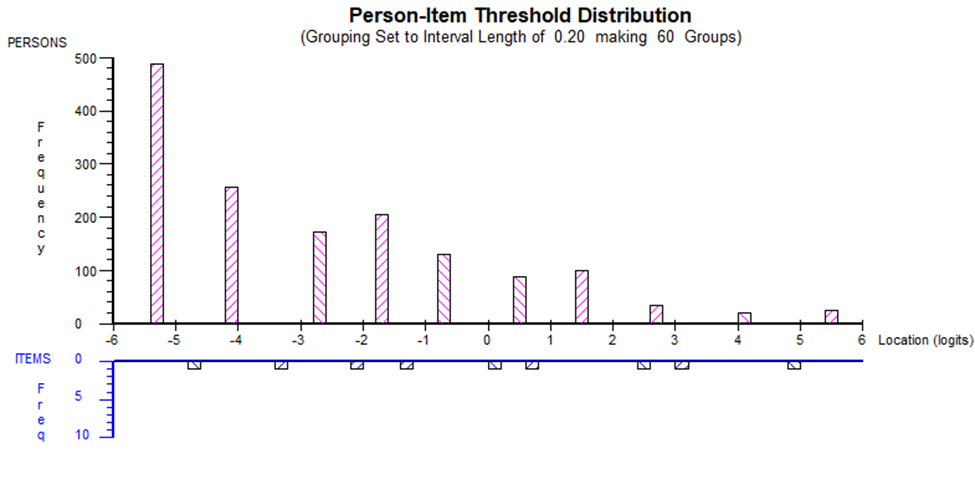
**

Figure 4: MG Symptoms Respiratory Muscle Weakness Targeting (all visits pooled) in the MG0003 study (Safety Set)

Legend: This figure shows the distribution of person measurements (upper histogram) against the distribution of the item threshold locations (lower histogram) on the Bulbar Muscle Weakness continuum. Here, the lower histogram (blue bars) shows the distribution of item thresholds which represent the boundaries between adjacent response categories.

**
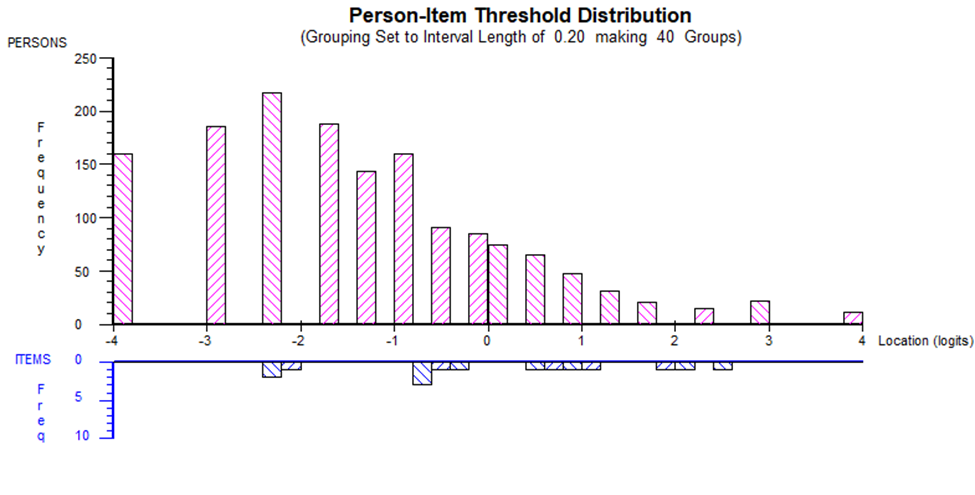
**

Figure 6: MG Symptoms Ocular Muscle Weakness Targeting (all visits pooled) in the MG0003 study (Safety Set) Legend: This figure shows the distribution of person measurements (upper histogram) against the distribution of the item threshold locations (lower histogram) on the Bulbar Muscle Weakness continuum. Here, the lower histogram (blue bars) shows the distribution of item thresholds which represent the boundaries between adjacent response categories.
