# Supplementary material for: Clinical meaningfulness and psychometric robustness of the MG Symptoms PRO scales in clinical trials in adults with myasthenia gravis
Source: Front Neurol. 2024 Jun 24;15:1368525. doi: 10.3389/fneur.2024.1368525 (PMC11229520; doi:10.3389/fneur.2024.1368525)
Supplement: Supplementary file 3 [file Data_Sheet_3.docx]

## Institutional Review Boards and Independent Ethics Committees

| **IRB/IEC** | **Number of study sites** | **Date(s) of IRB/IEC approval(s)** |
| --- | --- | --- |
| Advarra, 372 Hollandview Trail Suite 300, Aurora, Ontario, L4G 0A5, Canada | 14 | 15 May 2019  10 July 2019  19 July 2019  22 August 2019  25 September 2019  25 September 2019  10 October 2019  04 November 2019  11 December 2019  31 January 2020  05 February 2020  07 February 2020  14 February 2020  20 August 2020 |
| Biological Sciences Division, Institutional Review Board, 5841S Maryland Avenue, MC7132, I-625, Chicago, Illinois, 60637, USA | 1 | 26 November 2019 |
| Chiba University Hospital, 1-8-1 Inohana, Chuo-ku, Chiba-shi, Chiba, 260-8677, Japan | 1 | 23 March 2020 |
| Comitato Etico Agostino Gemelli, Università Cattolica del Sacro Cuore, Largo Agostino Gemelli 8, 00168 Roma, Italy | 1 | 28 October 2019 |
| Comitato Etico - AVEC, Via Albertoni 15, 40138 Bologna, Italy | 1 | 01 October 2020 |
| Comitato Etico Azienda Ospedaliera Cardarelli  Via Cardarelli 9, 80131 Napoli, Italy | 1 | 12 June 2020 |
| Comitato Etico dell’Università Sapienza, Via di Grottarossa, 1035-1039 c/o Azienda Ospedaliera Sant’Andrea, 00189 Roma, Italy | 1 | 19 December 2019 |
| Comitato Etico Fondazione IRCCS Istituto Neurológico Besta, Via Celoria 11, 20133 Milano, Italy | 1 | 01 October 2019 |
| Comitato Etico Pavia, Viale Golgi 19, 27100 Pavia, Italy | 1 | 31 January 2020 |
| CPP Sud-Méditerranée II, Hôpital Sainte Marguerite, Pavillon 9–1er étage, 270 Boulevard Sainte Marguerite, Marseille, 13274, France | 7 | 08 November 2019  08 November 2019  08 November 2019  08 November 2019  08 November 2019  10 January 2020  06 November 2020 |
| De Videnskabsetiske Komitéer for Region, Midtylland, Skottenborg 26, 8800 Viborg, Denmark | 4 | 06 September 2019  06 September 2019  06 September 2019  08 October 2020 |
| Ethics Committee of Serbia, Vojvode Stepe 458 Street, Belgrade, 11221, Serbia | 2 | 04 September 2020  04 September 2020 |
| Ethisch Comité UZA, Wilrijkstraat 10, 2650, Edegem, Belgium | 1 | 14 October 2019 |
| Ethikkommission an der Medizinischen Fakultät der Universität Leipzig, Liebigstr 18, 04103 Leipzig, Germany (local EC)  Ethikkommission der Friederich-Schiller-Universität Jena, Bachstraße 18, 07740 Jena, Germany (central EC) | 1 | 15 November 2019 |
| Ethikkommission der Ärztekammer Nordrhein, Tersteegenstraße 9, 40474 Düsseldorf, Germany (local EC)  Ethikkommission der Friederich-Schiller-Universität Jena, Bachstraße 18, 07740 Jena, Germany (central EC) | 1 | 20 January 2020 |
| Ethikkommission der Ärztekammer Westfalen-Lippe und der medizinischen Fakultät der WWU Münster, Gartenstr. 210–214, 48147 Münster, Germany (local EC)  Ethikkommission der Friederich-Schiller-Universität Jena, Bachstraße 18, 07740 Jena, Germany (central EC) | 1 | 15 November 2019 |
| Ethikkommission der Friederich-Schiller-Universität Jena, Bachstraße 18, 07740 Jena, Germany | 1 | 15 November 2019 |
| Ethikkommission der Medizinischen Fakultät der Universität Duisburg-Essen, Universitätsklinikum Essen, Robert-Koch-Str. 9–11, 45147 Essen, Germany (local EC)  Ethikkommission der Friederich-Schiller-Universität Jena, Bachstraße 18, 07740 Jena, Germany (central EC) | 1 | 15 November 2019 |
| Ethikkommission der Universitätsmedizin Göttingen, Von-Siebold-Straße 3, 37075 Göttingen, Germany (local EC)  Ethikkommission der Friederich-Schiller-Universität Jena, Bachstraße 18, 07740 Jena, Germany (central EC) | 1 | 25 February 2020 |
| Eticka Komise Všeobecná fakultní nemocnice v Praze, Na Bojisti 1, 128 08 Praha 2, Czech Republic | 1 | 17 October 2019 |
| Fakultni Nemocnice Ostrava, 17. listopadu 1790, 708 52 Ostrava, Czech Republic | 1 | 17 October 2019 |
| Fundació de Gestió Sanitària Hospital de La Santa Creu i Sant Pau, Sant Antoni Claret 167, Barcelona, 08025, Spain | 6 | 24 July 2019  24 July 2019  24 July 2019  04 December 2019  04 December 2019  29 January 2020 |
| General Hanamaki Hospital, 4-56, Otayacho, Hanamaki-shi, Iwate, 025-0082, Japan | 1 | 25 December 2019 |
| Hiroshima City Hiroshima Citizens Hospital  7-33, Motomachi, Naka-ku, Hiroshima, Hiroshima, 730-8518, Japan | 1 | 29 January 2020 |
| Houston Methodist Research Institutional Review Board, 6670 Bertner Avenue, Houston, Texas, 77030, USA | 1 | 24 September 2019 |
| Institutional Review Board of Shin Kong Wu Ho-Su Memorial Hospital, 95 Wen-Chan Road, Shih-Lin District, Taipei, 111, Taiwan | 1 | 13 February 2020 |
| Institutional Review Board of Taipei Veterans General Hospital, 201 Shih-Pai Road, Sec. 2, Taipei, 11217, Taiwan | 1 | 19 February 2020 |
| Juntendo University Hospital, 3-1-3 Hongo, Bunkyo-ku, Tokyo, 113-8431, Japan | 1 | 28 February 2020 |
| Keio University Hospital, 35 Shinano-Machi, Shinjuku-ku, Tokyo, 160-8582, Japan | 1 | 12 May 2020 |
| Kobe University Hospital, 7-5-2 Kusunoki-cho,  Chuo-ku, Kobe, Hyogo, 650-0017, Japan | 1 | 17 April 2020 |
| Komisja Bioetyczna przy UM, Al. Raclawickie 1, 059 Lublin, Poland | 4 | 19 December 2019  19 December 2019  19 December 2019  19 February 2020 |
| Local Ethics Committee of LTD Israeli-Georgian Medical Research Clinic Helsicore, 13 Tevdore Mgvdeli Street, Tbilisi, 0112, Georgia | 1 | 05 June 2020 |
| Local Ethics Committee of LTD New Hospitals, 12 Krtsanisi Street/71 Gorgasil Street, Tbilisi, 0114, Georgia | 1 | 05 June 2020 |
| Local Ethics Committee of LTD Petre Sarajishvili Institute of Neurology, 13 Tevdore Mgvdeli Street, Tbilisi, 0122, Georgia | 1 | 05 June 2020 |
| Local Ethics Committee of LTD Pineo Medical Ecosystem, 93 Gorgasali Street, Tbilisi, 0114, Georgia | 1 | 05 June 2020 |
| Local Ethics Committee of LTD Simon Khechinashvili University Hospital, 29-31-33 Chavchavadze Avenue, Tbilisi, 0179, Georgia | 1 | 05 June 2020 |
| Mayo Clinic, 201 Building, Room 4-60, 200 First St., SW Rochester, Minnesota, 55905, USA | 1 | 20 November 2019 |
| McGill University Health Center Research Board,  3801 University Street, Room 686, Montreal, Quebec, H3A 2B4, Canada | 3 | 18 March 2020  18 March 2020  18 March 2020 |
| Medical Research Council Ethics Committee for Clinical Pharmacology, Alkotmány u. 25, Budapest, 1054, Hungary | 2 | 25 September 2019  25 September 2019 |
| M.F. Vladimirsky Moscow Region, Ulitsa Shchepkina, 61/2, Moscow, 129110, Russia | 1 | 07 November 2019 |
| Nagasaki University Hospital, 1-7-1 Sakamoto, Nagasaki-shi, Nagasaki, 852-8501, Japan | 1 | 26 February 2020 |
| National Hospital Organization Sendai Medical Center, 2-11-12 Miyagino, Miyagino-ku, Sendai, Miyagi, 983-8520, Japan | 1 | 26 March 2020 |
| North-Western State Medical University, 41 Kirochnaya Ulitsa, Saint-Petersburg, 191015, Russia | 1 | 30 October 2019 |
| Oguz Novosibirsk State Region, 130 Nemirovicha-Danchenko Street, Novosibirsk, Novosibirsk Oblast, 630087, Russia | 1 | 16 July 2020 |
| Osaka University Hospital, 2-15 Yamadaoka Suita, Osaka, 565-0871, Japan | 1 | 25 February 2020 |
| Research Ethics Committee China Medical University & Hospital, 2 Yude Road, Taichung, 40447, Taiwan | 1 | 21 January 2020 |
| State Budgetary Institution, 2 Akkuratova Street, Saint-Petersburg, 197341, Russia | 1 | 17 February 2020 |
| State Budgetary Institution, 45 Prospekt Lunacharskogo, Saint-Petersburg, 194291, Russia | 1 | 21 February 2020 |
| Tokyo Medical University Hospital, 6-7-1, Nishi-Shinjuku, Shinjuku-ku, Tokyo, 160-0023, Japan | 1 | 12 May 2020 |
| University Health Network Research Ethics Board, 700 University Avenue, Hydro Building, 10/F Room 10-56, Toronto, Ontario, M5G 1Z5, Canada | 1 | 17 January 2020 |
| University of Alberta Health Research Ethics Board, 11313-98 Avenue NW, North Power Plant – West Entrance, Suite #2-01, University of Alberta North Campus, Edmonton, Alberta, T6G 2N2, Canada | 1 | 10 November 2020 |
| University of California Institutional Review Board, 1640 Marengo Street, Suite 700, Los Angeles, California, 90033, USA | 1 | 20 August 2019 |
| University Of Kentucky Medical Institutional Review Board, 315 Kinkead Hall, Lexington, Kentucky, 40506-0057, USA | 1 | 25 September 2019 |
| Wake Forest University Health Sciences, Medical Center Boulevard, Winston-Salem, North Carolina, 27157, USA | 1 | 28 October 2019 |
| Western Institutional Review Board, 1019 39th Avenue S.E. Suite 120, Puyallup, Washington, 98374, USA | 3 | 01 August 2019  03 September 2019  01 May 2020 |
| West Midlands - Edgbaston Research Ethics Committee, 3rd Floor Barlow House, Minshull Street, Manchester, M1 3DZ, United Kingdom | 1 | 14 November 2019 |

EC, Ethics Committee; IEC, Independent Ethics Committee; IRB, Institutional Review Board.
